# Efficacy of fluralaner spot-on solution for the treatment of *Ctenocephalides felis* and *Otodectes cynotis* mixed infestation in naturally infested cats

**DOI:** 10.1186/s12917-019-1775-2

**Published:** 2019-01-16

**Authors:** Antonio Bosco, Federico Leone, Rosachiara Vascone, Saverio Pennacchio, Lavinia Ciuca, Giuseppe Cringoli, Laura Rinaldi

**Affiliations:** 1 Department of Veterinary Medicine and Animal Production, University of Naples Federico II, Via Della Veterinaria 1, 80137 Naples, Italy; 2 Clinica Veterinaria Adriatica, Senigallia, Ancona, Italy; 3 Laboratorio di Analisi Veterinarie La Vallonea, Passirana di Rho, Milano, Italy

**Keywords:** Ectoparasiticides, Fleas, Ear mites, Cats

## Abstract

**Background:**

Cats can be infested with several ectoparasite species, especially *Ctenocephalides felis* and *Otodectes cynotis*. The aim of this study was to evaluate the efficacy of a single topical application of fluralaner against *C. felis* and *O. cynotis* natural infestation in stray (study 1) and owned (study 2) cats in central and southern Italy.

**Results:**

The number of live fleas found on each cat on Day 0 ranged from 1 to more than 30 (arithmetic mean live flea count = 11.9 in study 1; 14.6 in study 2) while no live fleas were found on days 7 and 84 post topical application of fluralaner. The number of live mites found on each cat on Day 0 ranged from 1 to 42 (arithmetic mean live mite count = 6.4 in study 1; 8.9 in study 2) while no live mites were found on days 7 and 84 post topical application of fluralaner.

**Conclusions:**

Topical fluralaner completely eliminated fleas and ear mites from infested cats and was 100% effective against both parasites up to 84 days after treatment.

## Background

Cats can be infested with several ectoparasite species, particularly *Ctenocephalides felis* and *Otodectes cynotis* that are responsible for dermatitis and ear infestation respectively [1, 2]. There are also some zoonotic pathogens such as *Rickettsia felis* and *Bartonella felis*, which can be transmitted by cat fleas [3]. In Europe the prevalence of flea infestations in cats ranges from 12% up to 70% [4, 5] as in Spain, Germany and Austria [6]. In Italy the prevalence ranges from 3.7 to 31.6% [6].

On the other hand *O. cynotis* is responsible for feline otodectic mange, in 50–80% of cases of *otitis externa* [7] and is present in up to 66% of these in cats [8, 9]. Ear mite infestation in cats may be complicated by secondary bacterial and fungal infection such as *Staphylococcus* spp. [10] and *Malassezia* spp. [9].

A multi-center study to determine the importance of *O. cynotis* revealed that it is the most prevalent ectoparasite of cats in Europe followed by *C. felis* [6]. In Italy, owned cats were found to be highly infested by these two ectoparasites, with values ranging from 31.6% for *O. cynotis* to 40.3% for *C. felis* [6].

Although there is high efficacy of antiparasitic molecules for the treatment and prevention of fleas [11] and mites [7, 12, 13] in cats, re-infestations with both species of ectoparasites occur frequently.

Therefore effective year-long control measures should be adopted as suggested by the European Scientific Council of Companion Animal Parasites [14].

Fluralaner spot-on formulation is an isoxazoline ectoparasiticide effective against arthropoda including ticks and fleas on cats [15]. In the field study by Meadows et al. [2], fluralaner was 99% effective against cat fleas from 4 to 12 weeks and 100% against *O. cynotis* during the 28-day observation period [15]. The aim of the present study was to evaluate the efficacy of a single treatment with a topical application of fluralaner against *C. felis* and *O. cynotis* natural infestations over 84 days.

## Results

All cats were positive for the presence of fleas and ear mites prior to Day 0 of the study. No adverse reactions related to topical administration of fluralaner were observed in any cat during the study. The number of live fleas found on each cat on Day 0 ranged from 1 to more than 30 (arithmetic mean live flea counts = 11.9 in study 1; 14.6 in study 2) while no live fleas were found up to day 84 (Table 1).

**Table 1:** *Ctenocephalides felis* and *Otodectes cynotis* counts (arithmetic mean and range) before (Day 0) and after (Days 7 to 84) a single topical dose of fluralaner in naturally infested cats and percent of efficacy

Number of cats	*Ctenocephalides felis*		*Otodectes cynotis*	
	Study 1	Study 2	Study 1	Study 2
	*n* = 14	*n* = 25	*n* = 14	*n* = 25
Mean parasite count	11.9	14.6	6.4	8.9
Parasite count range (Day 0)	1–30	1–30	1–21	1–42
Parasite count range (Days 7 to 84)	0	0	0	0
Percent efficacy^a^	100	100	100	100
*P* value	< 0.0001	< 0.0001	< 0.0001	< 0.0001

^a^From Day 7 to Day 84

The number of live mites found on each cat on Day 0 ranged from 1 to 42 (arithmetic mean live mite counts = 6.4 in study 1; 8.9 in study 2), while no live mites were found up to day 84 (Table 1).

At Day 0 of the study 1, 1–5 live fleas were recovered from the coat of 5 cats, 6–20 live fleas were found on 2 subjects, 1 cat was infested with more than 30 fleas and on 6 cats abundant flea faeces were found. In the study 2, 1–5 live fleas were recovered from the coat of 7 cats, 6–20 fleas were found on 5 subjects, 3 cats were infested with more than 30 fleas and in 10 cats abundant amounts of flea faeces were present.

On Day 0 in study 1, 10 cats had an otoscopic mite count of 1–5 mites, in the ears of 3 cats 6–20 mites were found and 1 cat was infested with more than 20 mites.

In the study 2, 11 cats had an otoscopic mite count of 1–5 mites, in 11 cats 6–20 mites were found and 3 cats had a severe mite infestation (more than 20 mites).

In both studies from Day 7 no mites or fleas were found on any cats. No ear mites were seen in situ in any cats following ear flushing. At each post-treatment assessment, the arithmetic mean flea/mite count reductions from baseline were significant (*P* <  0.0001).

The percentage efficacy of a single topical treatment with the spot-on solution of fluralaner against fleas and *O. cynotis* in naturally infested cats on Day 84 was calculated to be 100% based on arithmetic means.

The results of the pruritus/debris scoring are shown in Table 2. In both studies the severity of all clinical signs (pruritus on body and ears, head shaking, erythema, alopecia, excoriations, scratching and irritation at the ears) decreased over the course of the study starting as early as Day 7.

**Table 2:** Percentage of cats showing clinical pruritus and debris scoring on Day 0 (0 = absent, 1 = mild, 2 = moderate, 3 = sever/abundant)^a^

Score	Clinical pruritus scoring		Clinical debris scoring	
	Study 1	Study 2	Study 1	Study 2
0	42.8	0.0	0.0	0.0
1	35.7	0.0	0.0	8.0
2	7.1	40.0	7.14	28.0
3	14.2	60.0	92.8	64.0

^a^In both studies the severity of all clinical signs (pruritus, head shaking, erythema) decreased over the course of the study starting as early as Day 7 up to Day 84

## Discussion

A single topical dose of fluralaner completely eliminated fleas and ear mites in cats with mixed natural infestation by *C. felis* and *O. cynotis*, showing 100% efficacy against both ectoparasites at 84 days (12 weeks) after treatment.

Similarly to other ectoparasiticides of the isoxazolines class, fluralaner has a rapid action [16–19]; in our study, no fleas or mites were found 7 days after treatment. The complete control of both infestations lasted until the end of the study (day 84), the first time this has been shown for both species together making fluralaner a very valuable product for keeping cats free of infestations. Earlier studies had shown high activity of topical fluralaner against fleas for 12 weeks in naturally and experimentally infested cats [19, 20]. Furthermore, the efficacy of fluralaner against otodectic mites was previously reported in experimentally infested cats [1, 15]. In our present study, improvements in pruritus and otic clinical signs were observed after fluralaner treatment, and persisted up to Day 84.

In a similar study, the efficacy of afoxolaner in the treatment of natural infestations by *O. cynotis* in cats was shown but only for up to 35 days after the treatment [18].

In the present study there were no control groups, but the treated stray cats continued to live in close contact with other untreated infested cats during the trial so would have been exposed to flea (*C. felis*) and mite (*O. cynotis*) challenge.

## Conclusions

A single topical administration of fluralaner solution to cats is highly effective for controlling mixed flea and otodectic mite infestations, showing effective control for 3 months post-treatment.

## Methods

The study was conducted from February to July 2017 on stray (study 1) and owned (study 2) cats from two different geographical regions, Campania (southern Italy) and Marche (central Italy). Stray cats were recruited from a cattery located in the Campania region while the owned cats were recruited from two private clinics located in the Campania and Marche regions. This research was conducted with the approval of the Animal Care Committee of the Department of Veterinary Medicine and Animal Productions, University of Naples Federico II (protocol number 0041919/2017). All procedures were conducted in compliance with the ethical principles of good practice in animal experimentation.

### Animals

All animals were managed in the same way with regard to welfare and owner informed consent was obtained to use the cats. Enrollment criteria included cats of at least 11 weeks of age weighing at least 1.2 kg. None of the animals had been treated with acaricide or insecticide products for at least 12 weeks prior to this research.

### Study design and treatment

The study population consisted of 39 European breeds of cats (21 females and 18 males) divided in two groups (14 stray cats in study 1 and 25 owned cats in study 2), with weights ranging from 1.8 kg to 8.5 kg and between 1 and 8 years of age. Sixty days before treatment all cats were examined for clinical signs related to the presence of fleas and mites and suitability for the trial. All cats received a single dose (40 mg/kg) of fluralaner spot-on solution (Bravecto® spot-on, MSD Animal Health) on day 0. All treatments were applied by vets directly on the skin at the base of the skull, by squeezing the contents onto one or more spots according to the manufacturer’s instructions. Cats were checked for 24 h after dosing with follow-up performed at 7, 14, 28, 56 and 84 days to evaluate the effectiveness of fluralaner (Table 3).

**Table 3:** Design of the study

Study day	Event
Day − 60	• Inspection of the cattery • Clinical examination of the subjects
Day 0	• Application of fluralaner
Days 7, 14, 28, 56, 84	• Coat examination • Otoscopic examination • Cytological examination of the ear material • Clinical scoring^a^
Days 28, 56, 84	• Ear flushing

^a^Clinical scoring system. Pruritus (0 = absent, 1 = mild, 2 = moderate, 3 = sever); debris scoring (0 = absent; 1 = mild, 2 = moderate, 3 = abundant)

### Assessment of flea infestation

Prior to enrollment in the study, cats were proved to have existing flea infestations by a coat examination with a fine-toothed brush [21] and finding flea faeces and clinical examination for signs of infestation.

### Assessment of O. cynotis infestation

**Fig. 1:**
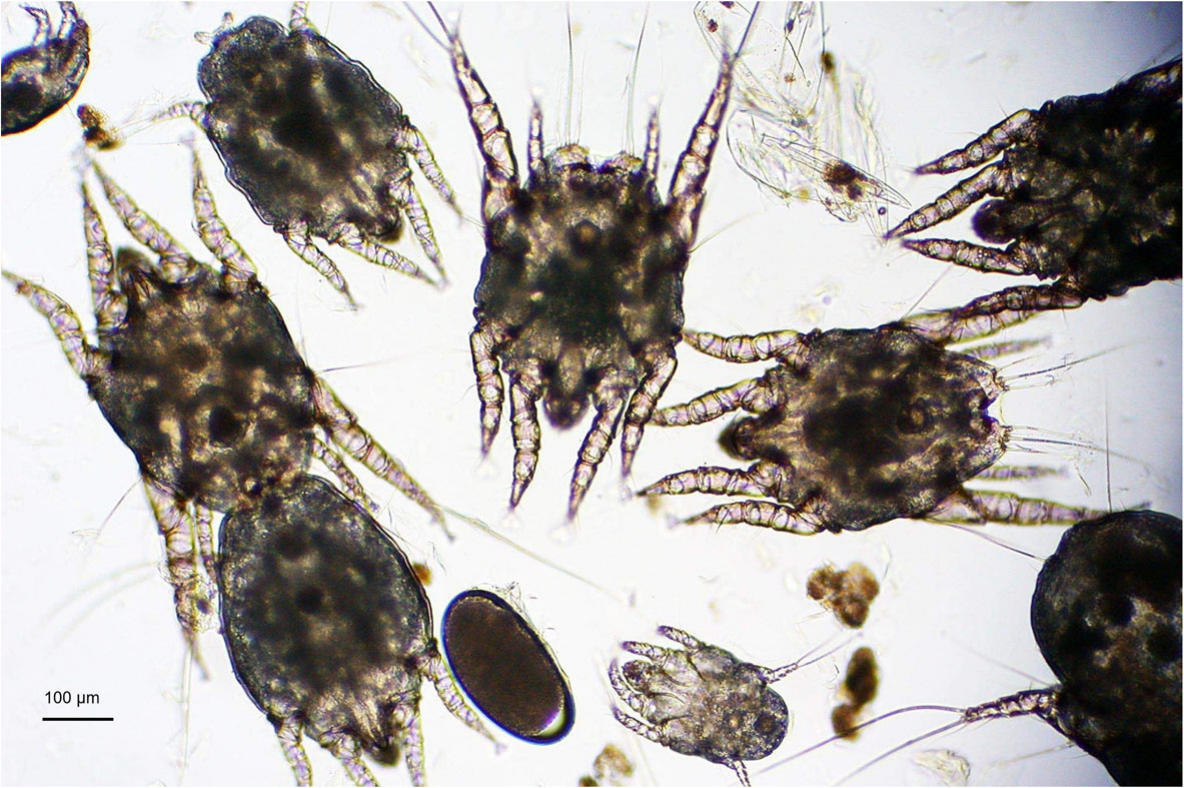
Microscopic examination of the auricular secretion of a cat infested by different stages of *O. cynotis*

Prior to enrollment, *O. cynotis* infestation was confirmed by direct or otoscopic examination of the external ear canal of both ears. On 28, 56 and 84 days post-treatment cats were sedated with dexmedetomidine hydrochloride (40 μg/kg IM), the ear ducts were filled with a solution of 0.9% sodium chloride (3 to 5 ml per ear canal).

and the ears were massaged externally to displace the content. The collected material was placed on slides and observed microscopically for immature and adult live mites (Fig. 1).

### Assessment of clinical signs

**Fig. 2:**
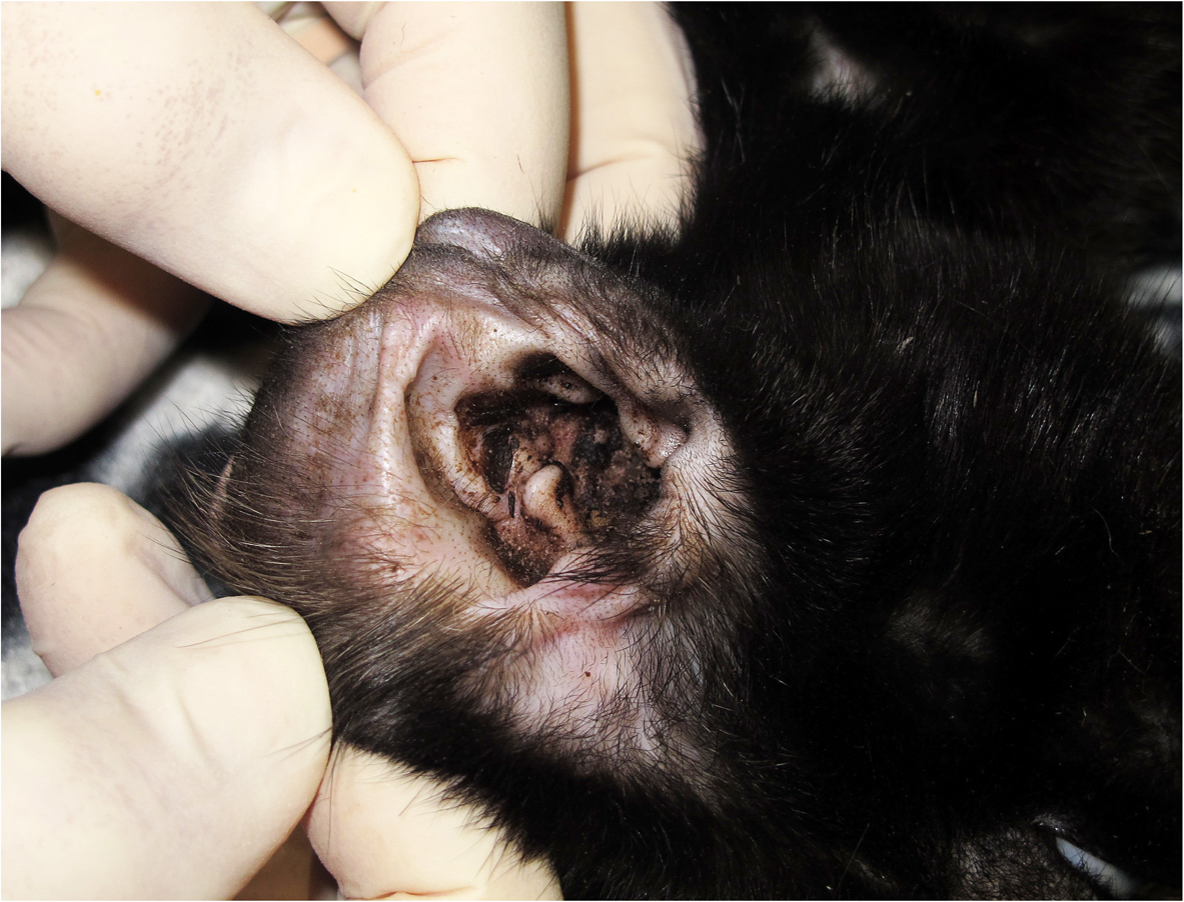
Auricular secretion with a characteristic “coffee ground” appearance in a cat affected by otoacariosis

**Fig. 3:**
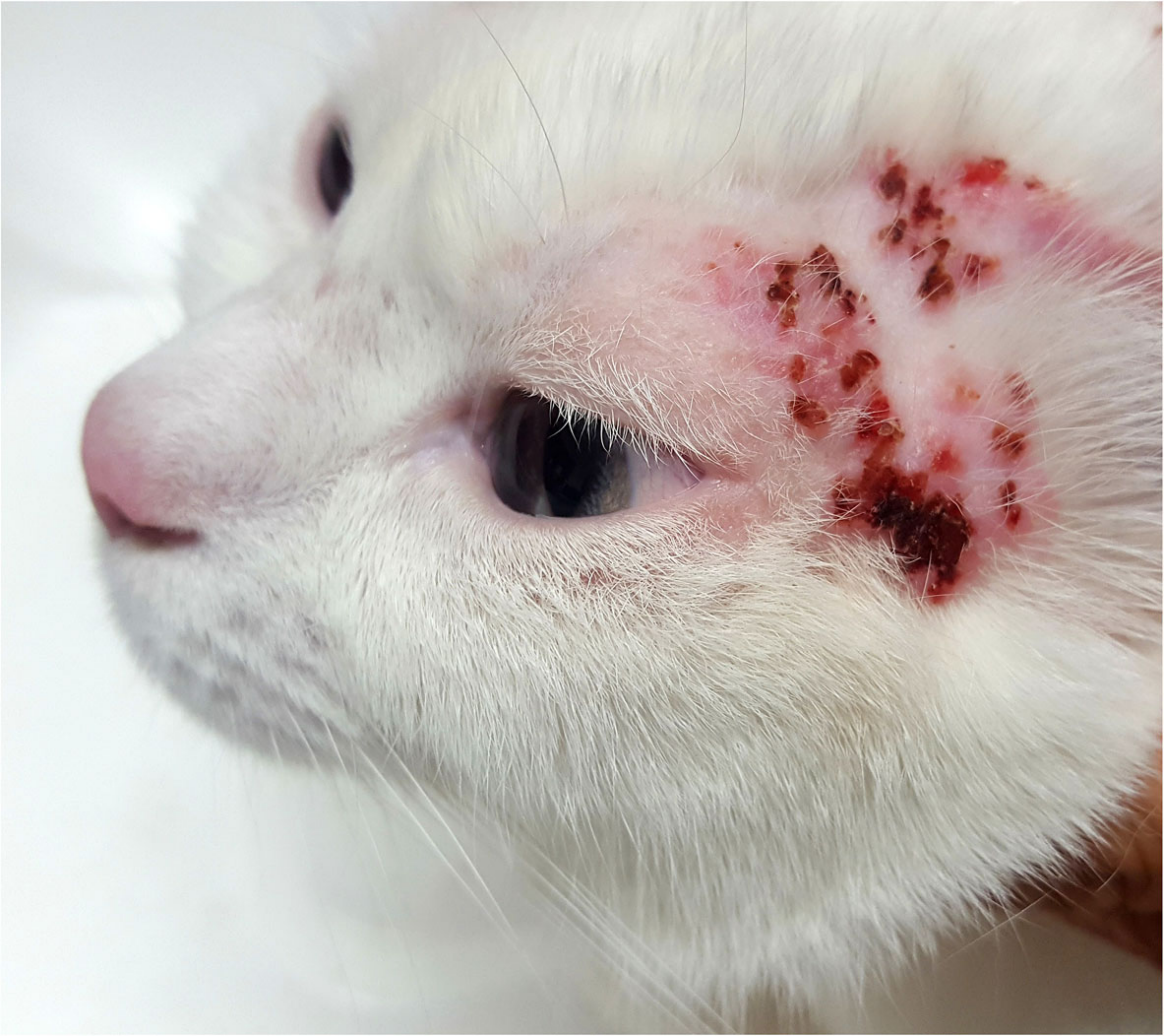
Self-traumatism injuries due to *Otodectes cynotis* in an infested cat

The clinical signs of infested cats before the treatment varied in severity from one cat to another and included pruritus, alopecia, papules, scales, crusts, excoriations, head shaking, erythema, ulceration and debris in the ear canal (Figs. 2 and 3).

Furthermore, during the clinical visit a score (Table 3) for each cat was awarded for pruritus (0 = absent, 1 = mild, 2 = moderate, 3 = severe) as described by Meadows et al. [2] and for ear debris (0 = absent; 1 = mild, 2 = moderate, 3 = abundant) [22].

### Efficacy evaluation

In both studies, the primary efficacy criterion was the number of live fleas/mites (in all stages) collected from the two groups (stray and owned cats) from 0 to 84 days post treatment. The average percent reduction in the flea/mite count was calculated using the following formula (adapted from Marchiondo et al. [23]):

$$ \%\kern0.5em \mathrm{Efficacy}=\frac{\mathrm{Mean}\ \mathrm{F}/\mathrm{M}\ \mathrm{Day}\ 0-\mathrm{F}/\mathrm{M}\ \mathrm{Day}\mathrm{s}\ 7,14,28,56,84}{\mathrm{Mean}\ \mathrm{F}/\mathrm{M}\ \mathrm{Day}\ 0}\times 100 $$

where:

F = mean live flea count;

M = mean live mite count.

After the study, all the cats involved in the two trials (study 1 and study 2) returned to their normal living conditions (cattery or home) and received regular veterinary care.

## Abbreviations

F: Mean live flea count
M: Mean live mite count
